# Comparison of the gut microbiome of sacbrood virus-resistant and -susceptible *Apis cerana* from South Korea

**DOI:** 10.1038/s41598-022-13535-0

**Published:** 2022-06-15

**Authors:** Bo-Ram Yun, A-Tai Truong, Yong Soo Choi, Man Young Lee, Byoung Yong Kim, Minjung Seo, Soon-Seek Yoon, Mi-Sun Yoo, Dong Van Quyen, Yun Sang Cho

**Affiliations:** 1 Parasitic and Insect Disease Laboratory, Bacterial Disease Division, Department of Animal and Plant Health Research, Animal and Plant Quarantine Agency, Gimcheon, 39660 Republic of Korea; 2 Department of Agricultural Biology, National Institute of Agricultural Science, Wanju, 55365 Republic of Korea; 3 ChunLab Inc., Seoul, 06194 Republic of Korea; 4 University of Science and Technology of Ha Noi, Vietnam Academy of Science and Technology, 18 Hoang Quoc Viet, Cau Giay, Hanoi, Vietnam; 5 Division of Vectors and Parasitic Diseases, Korea Disease Control and Prevention Agency, 187 Osongsaengmyeong 2-ro, Heungdeok-gu, Cheongju, Chungbuk 28159 Republic of Korea; 6 Department of Veterinary Medicine, College of Veterinary Medicine, Kyungpook National University, Buk-gu, Daegu, 41566 Republic of Korea; 7 Faculty of Biotechnology, Thai Nguyen University of Sciences, Thai Nguyen, 250000 Vietnam

**Keywords:** Infectious diseases, Viral infection, Diseases, Microbiology, Microbial communities, Microbiome

## Abstract

Honey bees are important pollinators for the conservation of the ecosystem and agricultural products and provide a variety of products important for human use, such as honey, pollen, and royal jelly. Sacbrood disease (SD) is a devastating viral disease in *Apis cerana*; an effective preventive measure for SD is urgently needed. In this study, the relationship between the gut microbiome of honey bees and SD was investigated by pyrosequencing. Results revealed that sacbrood virus (SBV)-resistant *A. cerana* strains harbour a unique acetic acid bacterium, *Bombella intestini*, and the lactic acid bacteria (LAB) *Lactobacillus* (unclassified)_uc*, Bifidobacterium longum, B. catenulatum, Lactococcus lactis,* and *Leuconostoc mesenteroides* in larvae and *Hafnia alvei, B. indicum,* and the LAB *L. mellifer* and *Lactobacillus* HM215046_s in adult bees. Changes in the gut microbiome due to SBV infection resulted in loss of bacteria that could affect host nutrients and inhibit honey bee pathogens, such as *Gilliamella* JFON_s, *Gilliamella_*uc*, Pseudomonas putida,* and *L. kunkeei* in *A. cerana* larvae and *Frischella*_uc*, Pantoea agglomerans*, *Snodgrassella*_uc*,* and *B. asteroides* in adult bees. These findings provide important information for the selection of probiotics for *A. cerana* larvae and adults to prevent pathogenic infections and keep honey bees healthy.

## Introduction

Honey bees are important insects acting as pollinators as well as producers of various beneficial products, such as honey, propolis, pollen, royal jelly, and bee venom^1,2^. Sacbrood virus (SBV) causes serious damage to the honey bee industry, especially to *Apis cerana*, in Southeast Asian countries including Thailand and Vietnam and East Asian countries such as Taiwan, China and Korea^3–7^. In Korea, SBV was first detected in 2008^8^, and since 2009, sacbrood disease (SD) has resulted in severe losses in Korean apiaries due to colony collapse disorders, similar to that observed in other Southeast Asian countries and China in 1970s^9,10^; SD continued to be the highest economic loss-causing disease in *A. cerana* apiaries even in 2021. Therefore, for the development of SBV-resistant honey bee strains, there is a need for research and development of various preventive measures, such as probiotics, to conserve *A. cerana* and preserve species-diversity among honey bees.

In a previous study, surviving strains of *A. cerana* from SD-affected apiaries were collected, and breeding techniques were applied to produce two lines of *A. cerana* with resistance to SBV^11^, R (with individual immunity) and H (with social immunity). Hybridisation of these two lines was demonstrated to confer high resistance to SBV in South Korea by increasing hygienic behavior and brood survival rate^12^. Furthermore, SBV is known to exhibit high-affinity binding to the gut of honey bees^4,13^, and the gut microbiota may influence SBV infection and SD progression^14–18^. Therefore, in this study, guts from SBV-resistant and -susceptible *A. cerana* were collected and identification of the microbiome was performed by pyrosequencing. The differences in the microbiota of SBV-resistant and -susceptible strains of *A. cerana* could provide helpful information on SBV resistance in this species, which could prove helpful in the development of efficient candidates for honey bee probiotics for SD.

## Results

### Gut microbiome of SBV-susceptible *A. cerana*

**Figure 1:**
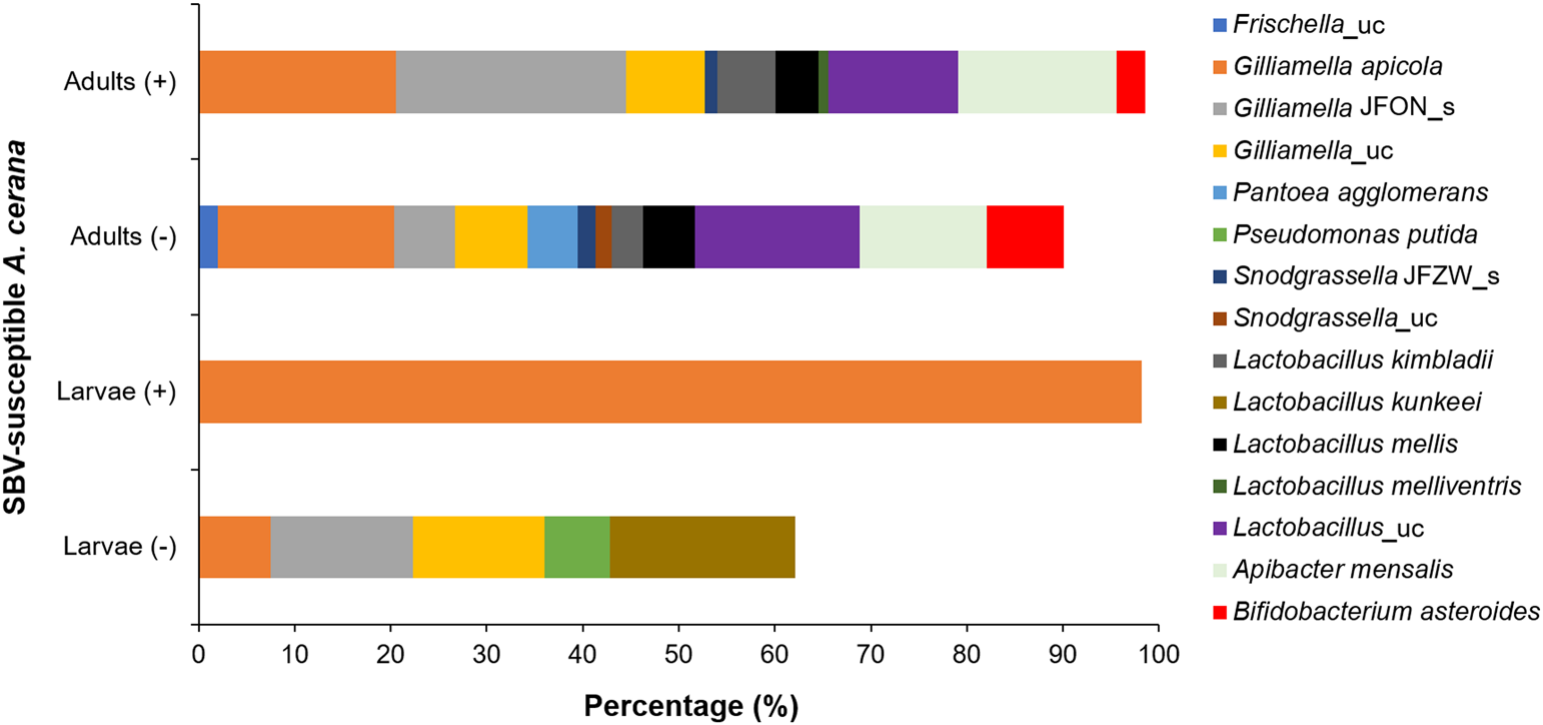
Gut microbiome of SBV-susceptible *Apis cerana*. Bacterial species were identified from the gut of sacbrood virus (SBV)-susceptible *A. cerana* adults and larvae from both healthy (−) and sacbrood disease (SD)-affected (+) honey bees collected from Cheongju province. (“uc” indicates unclassified species; “JFON_s” and “JFZW_s” indicate uncultured species of genera *Gilliamella* and *Snodgrassella*, respectively).

The gut microbiota prevalent in healthy larvae of SBV-susceptible *A. cerana* included the phyla *Proteobacteria* and *Firmicutes*, genera *Gilliamella*, *Pseudomonas*, *Lachnospiraceae*_uc, and *Lactobacillus*, and species *L. kunkeei* (19.29%), *Gilliamella* JFON (uncultured species) (14.81%), *Gilliamella*_uc (13.71%), *G. apicola* (7.51%), and *Pseudomonas putida* (6.81%) (Fig. 1; Table S1). SBV infection resulted in the decline of bacterial diversity in *A. cerana* larvae (*p* = 0.0001). The gut microbiome of SD-affected larvae was found to predominantly consist of *G. apicola* (98.20%) and only a minute percentage of the LAB *L. kimbladii* (0.02%) (Fig. 1; Tables S1 and S2).

The diversity and abundance of the gut microbiome in adult honey bees were higher than those in larvae. The gut microbiota prevalent in *A. cerana* adults of SBV-susceptible colonies included the phyla *Proteobacteria* (43.04%), *Firmicutes* (25.76%), *Bacteroidetes* (13.24%), and *Actinobacteria* (8.08%), genera *Frischella*, *Gilliamella*, *Pantoea, Snodgrassella*, *Lactobacillus*, *Apibacter,* and *Bifidobacterium*, and species *G. apicola* (18.35%), *P. agglomerans* (5.25%), *Frischella*_uc (1.97%), *Snodgrassella* JFZW (uncultured species) (1.84%), *A. mensalis* (13.24%), and LAB—*B. asteroides* (8.08%), *L. kimbladii* (3.27%), and *L. mellis* (5.35%) (Fig. 1; Table S1). Species composition of the gut microbiota in diseased *A. cerana* adults was not notably changed in comparison with that in healthy adults (*p* = 0.3103); the changes were identified by an increase in the abundance of *Gilliamella* JFON_s, *L. kimbladii,* and *A. mensalis*, slight decrease in the abundance of *L. mellis, Lactobacillus*_uc, and *B. asteroides*, and the loss of *Frischella*_uc and *P. agglomerans* (Fig. 1; Table S1).

### Gut microbiome of SBV-resistant *A. cerana*

**Figure 2:**
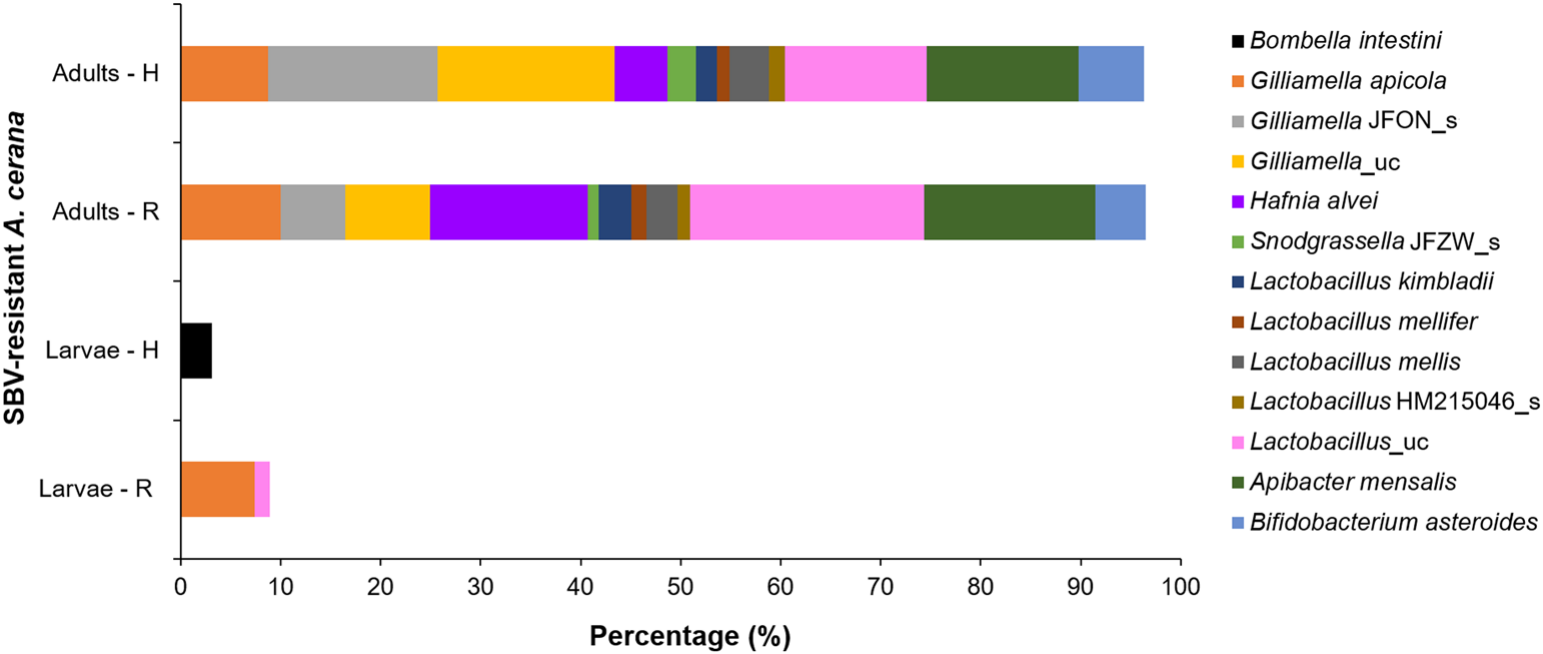
Gut microbiome of SBV-resistant *A. cerana*. The gut microbiota of adults and larvae of two artificially bred *A. cerana* strains, R (individual immunity) and H (social immunity), was identified and confirmed to be capable to resist SBV (“uc” indicates unclassified species; “JFON_s” and “JFZW_s” indicate uncultured species of genera *Gilliamella* and *Snodgrassella*, respectively; HM215046_s indicates the NCBI accession number of *Lactobacillus* species).

The gut microbiota of SBV-resistant *A. cerana* larvae of R and H strains belonged to only two phyla, *Proteobacteria* and *Firmicutes*. The gut microbiota of SBV-resistant larvae showed a lower diversity than that of SBV-susceptible larvae. The difference of gut microbiota was significant between SBV-resistant H strain and SBV-susceptible strain (*p* = 0.0001). The major species in the R strain were *G. apicola* and *Lactobacillus*_uc, and only *Bombella intestini* was identified in the H strain (Fig. 2; Table S1).

The gut microbiota prevalent in SBV-resistant *A. cerana* adults in R and H strains comprised the same phyla, namely *Proteobacteria*, *Firmicutes*, *Bacteroidetes,* and *Actinobacteria*. Further, gut microbiota of SBV-resistant strains was not significantly different from that of susceptible strains, *p* = 0.6839 and 0.5862 between susceptible strains and resistant R strain and between susceptible strains and resistant H strain, respectively. In comparison with the gut microbiome of SBV-susceptible strains, SBV-resistant strains showed three new species, *H. alvei*, *L. mellifer,* and *Lactobacillus* HM215046_s, and lacked *Frischella*_uc and *P. agglomerans*. In addition, *Gilliamella* JFON_s (17.01%) and *Gilliamella*_uc (17.66%) were more abundant in the resistant H strain than in the resistant R strain exhibiting *Gilliamella* JFON_s and *Gilliamella*_uc at 6.45% and 8.48%, respectively, and those in the susceptible strains were 6.37% and 7.53%, respectively. *G. apicola* was dominantly detected in the susceptible strain (18.35%). However, it was detected at only 9.98% and 8.71% in R- and H-resistant strain, respectively (Fig. 2; Table S1).

### Lactic acid bacteria in *A. cerana*

**Figure 3:**
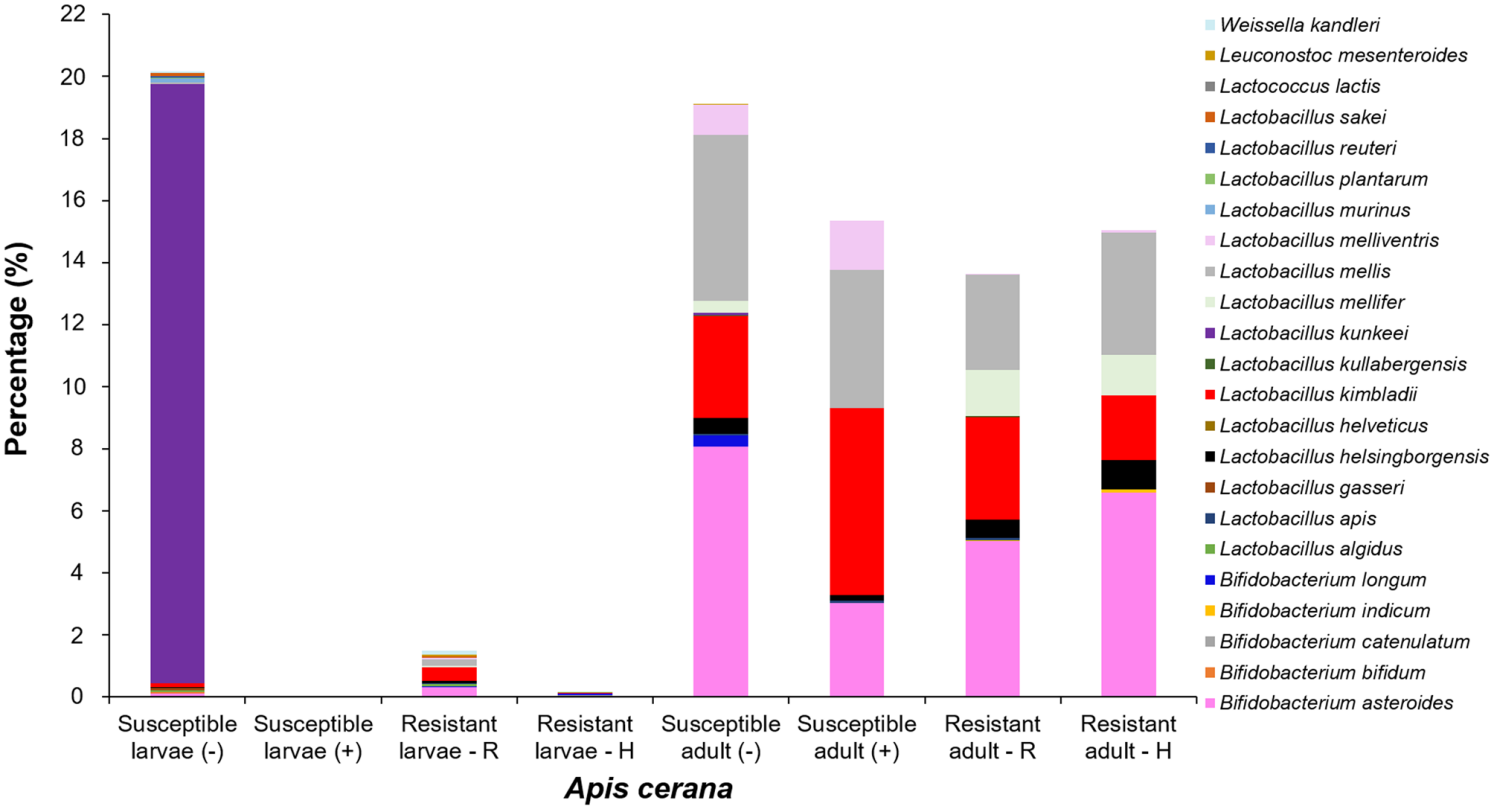
Lactic acid bacteria (LAB) in the gut of *A. cerana*. LAB were identified from the gut of adults and larvae of SBV-susceptible and -resistant strains (R and H) of *A. cerana*. The (−) and (+) symbols indicate healthy and SD-affected honey bees, respectively.

The LAB species *L. kunkeei* (19.29%) was predominant in the gut of SBV-susceptible *A. cerana* larvae, while *B. asteroides* (8.08%), *L. mellis* (5.35%), and *L. kimbladii* (3.27%) were identified in the gut of SBV-susceptible adults. There was a major loss of LAB in the gut of SBV infected larvae; only one LAB species, *L. kimbladii* (0.022%), was detected in the diseased larvae. Similarly, in diseased adults, loss of the LAB species *B. longum*, *L. kullabergensis*, *L. kunkeei* and *Leuconostoc mesenteroides* and a decrease in the abundance of *B. asteroides* and *L. mellis* to 3.02% and 4.44%, respectively, were observed. However, the abundance of *L. kimbladii* and *L. melliventris* in diseased adults increased from 3.27% and 0.98% to 6.03% and 1.59%, respectively (Fig. 3; Table S2).

Although the number of LAB in the larvae of SBV-resistant *A. cerana* was lower than that in susceptible larvae, the difference was not remarkable. *B. longum* was present in both R and H strains. LAB identified in larvae of only the R strain were *L. apis, L. mellifer*, *L. melliventris*, *Lactococcus lactis,* and *Leuconostoc mesenteroides*, and LAB in larvae of only the H strain comprised *B. catenulatum*. There was no notable difference between the SBV-susceptible and -resistant adults in terms of species number and abundance of LAB (Fig. 3; Table S2).

### Diversity analysis of gut bacterial communities

**Figure 4:**
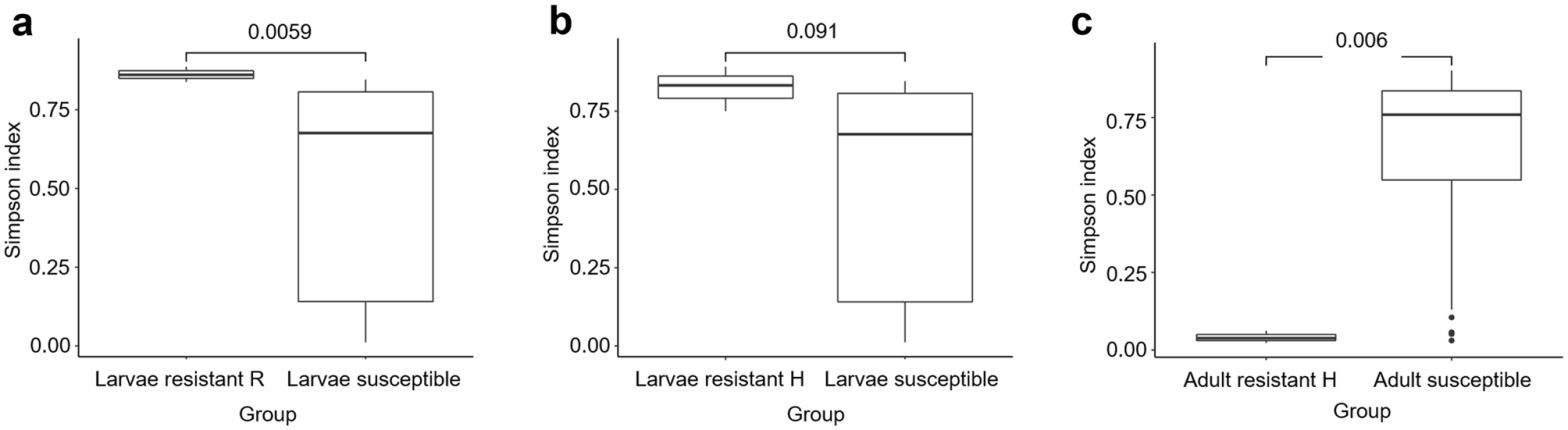
Alpha diversity boxplots designed with Simpson index was used to compare the gut microbiome between SBV-resistant and -susceptible honey bee strain. The comparison of microbiome between larvae of resistant strain R and that of susceptible strain (**a**), and between resistant strain H and susceptible strain (**b**). The difference in gut microbiome between adult bee of resistant train H and susceptible strain was also shown (**c**).

**Figure 5:**
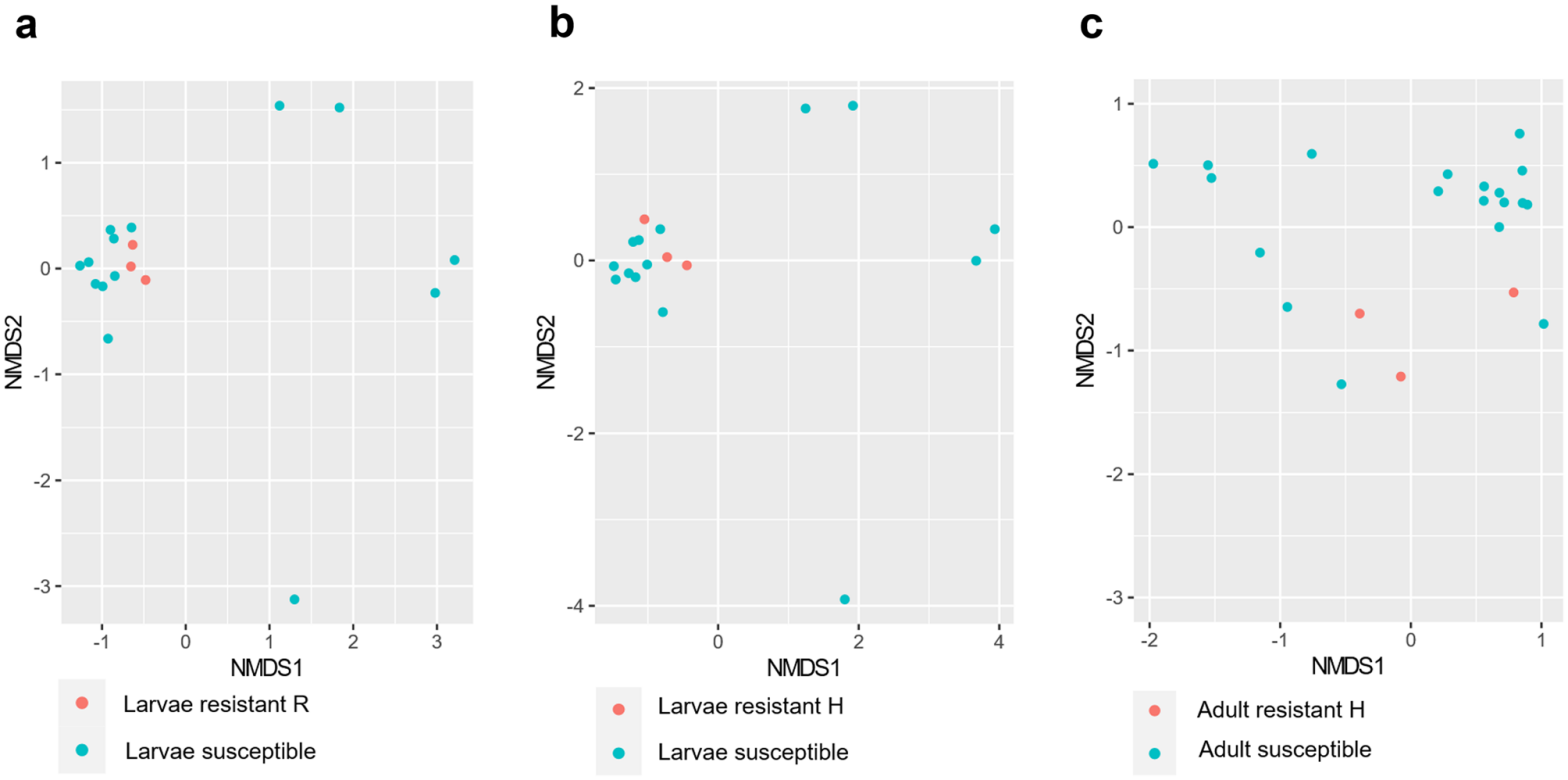
Gut bacterial communities of larvae and adult bee of SBV-resistant strain compared to SBV-susceptible strain were analysed by the non-metric multidimensional scaling (NMDS). The analysis was done for comparison of microbiome of SBV-resistant larvae strain R (**a**) and strain H with SBV-susceptible strain (**b**), and of adult bee of resistant strain H and susceptible strain (**c**).

The difference in biodiversity of gut microbiome between SBV-resistant and SBV-susceptible honey bee was shown by alpha diversity with Simpson parameters. The *p*-value of diversity index between the larvae of SBV-resistant R strain and susceptible strain was 0.0059 (Fig. 4a), and between resistant H strain and susceptible strain was 0.091 (Fig. 4b). Meanwhile, the *p*-value of comparison between resistant adult and susceptible adult was 0.006 (Fig. 4c). Furthermore, the gut bacterial community in larvae of SBV-resistant strain and susceptible strain (Fig. 5a,b), and in adult bee of the two strains (Fig. 5c) can be distinguished using non-metric multidimensional scaling (NMDS) based on Bray–Curtis indices. The NMDS stress value of resistant larvae strain R and H compare to susceptible strain was 0.0978 and 0.0979, respectively, and the value of adult bee comparison was 0.1059. Alpha diversity and NMDS of other comparisons were shown in Figs. S1, S2, and S3.

## Discussion

In this study, we found differences in the microbiota of SBV-susceptible and -resistant *A. cerana* in Korea for the first time. The microbiota of SBV-resistant *A. cerana* larvae included *Bombella intestini* and *Lactobacillus*_uc, which were absent in SBV-susceptible *A. cerana* larvae. Members of the core gut community in honey bees have been known to be *S. alvi* (class: Betaproteobacteria; family: Neisseriales), *G. apicola*, *F. perrara* (Gammaproteobacteria; Orbales), Alphaproteobacteria, and Lactobacillae (Firmicutes; Lactobacillaceae)^19^. *Acinetobacter* sp., *Fructobacillus fructosus*, *Commensalibacter intestine,* and *L. kimbladii* have been identified in the gut microbiome of *A. mellifera* in a previous study^19–22^. Thus, *Bombella intestini* was a species unique to SBV-resistant *A. cerana*, when compared with *A. mellifera* and SBV-susceptible *A. cerana*. *Bombella intestini* is an endosymbiotic acetic acid bacterium found in bumble bees (*Bombus bimaculatus*)^23,24^, which was also identified in *A. cerana* for the first time in this study.

SBV has been known as a gut-affinity virus, leading to damage of the gut of larvae and subsequent rotting of the diseased larvae^6^. One of the preventive measures for honey bee diseases is probiotics, which provide nutrients, protect the attachment of pathogens to cell surfaces, and create an acidic environment that is harsh for the survival of pathogens^20,25–27^. Therefore, probiotics could have positive effects on the survival of SBV-infected *A. cerana*. The unique microbiota in SBV-resistant adults in comparison with the SBV-susceptible adults included *H. alvei* and the LAB *L. mellifer*, *Lactobacillus* HM215046_s, and *B. indicum*. *H. alvei* was assumed to be an opportunistic pathogen of honey bees^28^. However, the function of this bacterium in SBV-resistant adult bees remains unclear. Besides, LAB unique to the larvae of SBV-resistant *A. cerana* compared to those of susceptible strains were *B. catenulatum*, *B. longum*, *L. apis*, *L. mellifer*, *L. melliventris*, *Lactococcus lactis,* and *Leuconostoc mesenteroides*. LAB produce organic acids, known as anti-microbial metabolites, inhibiting the growth of spoilage and pathogenic microorganisms^29^. Therefore, microbiota unique to SBV-resistant *A. cerana* in each developmental stage could be useful for the development of probiotics for disease prevention in honey bee larvae and adults.

LAB play important roles in the production and preservation of honey bee nutrients^30^. In addition, several studies have showed that LAB was helpful in increasing the size of honey bee colony by increasing the egg-laying capacity of the queen^31,32^ and resistance to honey bee diseases such as nosemosis^31,33,34^ and varroosis^31^. Common LAB in all the larvae and adults of *A. cerana* in this study were *B. asteroides*, *L. helsingborgensis*, *L. kimbladii* and *L. mellis*, of which *L. helsingborgensis* and *L. kimbladii* were also isolated from *A. mellifera* and described for the first time in 2014^22^. They showed the ability to produce acid from d-glucose, d-fructose, d-mannose, *N*-Acetylglucosamine, arbutin, salicin, and d-tagatose^22^. However, further studies on the usefulness of the LAB species unique to SBV-resistant *A. cerana* might be important to understand whether they provide practical resistance against SBV infection.

Comparison of the gut microbiota of healthy and SD-affected larvae in SBV-susceptible *A. cerana* revealed that SD progression resulted in the loss of *Gilliamella* JFON_s, *Gilliamella*_uc, *Pseudomonas putida,* and *L. kunkeei*. Some of these bacterial species have been identified to have important functions in the gut of honey bees. For instance, *Gilliamella* spp. are endosymbionts and play a role in degrading polysaccharides that could affect the absorption of host nutrients^35^, *Pseudomonas putida* has the ability to degrade neonicotinoid insecticides^36^, and *L. kunkeei* is known to inhibit opportunistic pathogens^37^. Furthermore, SBV infection in adult bees resulted in the loss of *Frischella*_uc, *P. agglomerans*, *Snodgrassella*_uc, *B. longum*, *L. kullabergensis,* and *L. kunkeei* and a decrease in the abundance of *B. asteroides* and *L. mellis* in the gut*. Frischella* spp., such as *F. perrara*, is known to stimulate the immune system of *A. mellifera*^38^, and *P. agglomerans* acts as a biocontrol agent against fire blight and human facultative pathogens^39^. *G. apicola*, *L. kimbladii* and *B. asteroides* produce acidic products via fermentation^40,41^. *G. apicola* was also demonstrated to have the ability to break down various carbohydrates that are potentially toxic to honey bees^42^. *A. mensalis, Snodgrassella* spp., and *F. perrara* were found to stimulate the immune system of *A. mellifera*^38^. However, the mutualistic interaction between the host and the *Apibacter* spp. remained unclear^43,44^. Other LAB species are capable of digesting polysaccharides and producing bioactive compounds that possess the potential to act as antimicrobials^35,40,45^. Therefore, supplements of these bacteria as probiotics could be helpful to maintain a healthy gut environment and provide efficient protection against SD.

There was no common essential microbiota in the larvae. However, in adults, the common microbiota between susceptible and resistant strains were seen; these included *G. apicola*, *Gilliamella* JFON_s, *Gilliamella*_uc, *Snodgrassella* JFZW_s, *L. kimbladii*, *L. mellis, A. mensalis,* and *B. asteroids*. The common microbiota in adults are vital for survival^46^. Evidence suggests that differences in the gut microbiota could have originated from differences in the natural environment and the queen lineages of honey bees^46–50^. The comparison of the gut microbiome from healthy, susceptible adult honey bees collected from two provinces, Jeju and Cheongju, showed that the major bacterial species present in the gut of adults were not notably different (*p* = 0.493). However, the diversity of the gut microbiome in adults was higher in Cheongju than in Jeju (Fig. S4a). Interestingly, considerable differences were observed in the gut microbiome of larvae collected from the two provinces (*p* = 0.0436; Fig. S4b). There were also differences observed in the gut microbiome collected in different seasons. The identification of the gut microbiome collected in June and October in Cheongju province was not significantly different, *p* = 0.5802 and 0.2039 for adult and larvae, respectively. The results revealed that *P. agglomerans* was predominant in adults in October, while *G. apicola* was predominant in June (Fig. S5a). In case of the larval gut, *L. kunkeei*, *Gilliamella*_uc, and *Snodgrassella*_uc were found in October and *Gilliamella*_uc and *G. apicola* in June (Fig. S5b). Therefore, further studies are required to understand the influence of environmental or natural factors, such as type of pollen and nectar in sampling sites, on the actions of gut bacteria in honey bees and interaction between the host and the bacteria.

In conclusion, the gut microbiota unique to SBV-resistant *A. cerana* was identified. This study revealed that the SBV infection resulted in the loss of gut microbiota that could affect host nutrients and inhibit certain opportunistic pathogens in *A. cerana*. The results of this study can provide important information for designing and developing developmental stage- and strain-based probiotics that could be formulated including the essential common bacterial group, specific species in resistant strains, and the important common LAB group. The probiotics could be important to protect *A. cerana* from pathogenic infections, and for further research on preventing severe SD outbreaks and economic losses to apiaries.

## Materials and methods

### Selection of apiaries

Three apiaries of SBV-susceptible *A. cerana* and one of SBV-resistant *A. cerana* were selected in the regions with 4 clear seasons. Two of these apiaries were free of SD and located in Cheongju and Jeju, at coordinates 36°31′13.1″ N–127°29′29.7″ E, and 33°15′50.3″ N–126°19′53.9″ E, respectively. The third apiary was SD-inflicted and located in Cheongju (35°30′11.7″ N–127°28′16.1″ E) (Table 1), and the fourth one with two SBV-resistant colonies was located in Wanju (35°90′39″ N–127°16′22″ E). Collection of SD-free samples from Cheongju was done in June and October and in March for SD-affected ones. SD-free honey bees from Jeju were collected in June, and SBV-resistant samples from Wanju were collected in May. The two lines of SBV-resistant *A. cerana*, developed by the National Institute of Agricultural Science, were the R strain with individual immunity against SBV infection and H strain with social immunity against SBV infection. All the colonies of sample collection were confirmed with free of other pathogens using the LiliF™ SBV/KSBV/DWV/BQCV reverse transcription, real-time polymerase chain reaction (RT-qPCR) Kit, LiliF™ ABPV/KBV/IAPV/CBPV RT-qPCR Kit (iNtRON Biotechnology, Inc., Seongnam, Korea), and the POBGEN™ Bee Pathogen Detection Kits (DB-A2 and DB-B2) (POSTBIO Inc., Guri, Korea).

**Table 1:** Characteristics and sacbrood disease status of honey bee samples for gut microbiome analysis.

Characteristics	Region	Sacbrood disease	SBV resistance	Collection time	Hives (*n*)	Larvae (*n*)	Adults (*n*)
SBV susceptibility	Cheongju	−	−	June	6	11	18
				October	4	4	8
	Jeju	−	−	June	1	1	5
	Cheongju	+	−	March	3	5	5
SBV resistance, H strain	Wanju	−	+	May	1	3	3
SBV resistance, R strain					1	3	3
	Total				16	27	42

SD colonies were identified by clinical signs, which were the irregular capping of combs, the pulled-out larvae out of hives, shrunk or rotten larvae, and larvae inclined to the cell wall. The presence of SBV in larvae and adult bees was confirmed by SBV detection by real-time PCR using specific primers (forward primer: 5′-AGA AGT TTT GGT GTA TAT GCG AGG-3′ and reverse primer: 5′-CTG CGC AGT TTC ATC TTC ATC TTC-3′, and probe 5′-HEX-AAA TAG ACC AAG AAG GGA ATC AGA TAA TCC-BHQ-1-3′)^10^.

### Collection of samples

Larvae and adults of SBV-susceptible and -resistant *A. cerana* were collected and transported to the laboratory in refrigerated conditions (4 °C). Guts were isolated from both adults and larvae and stored in Eppendorf tubes at − 20 °C before sending them for microbiome analysis by pyrosequencing. Number of larvae and adults collected from each colony varied from one to three depending on the quality of extracted gut and sequencing result. Information of collected samples is shown in Table 1.

### Extraction of nucleic acid

The collected gut samples were added to Lysing Matrix E tubes containing ceramic beads (MP Biochemicals GmbH, Eschwege, Germany). After adding PBS (400 μl) to the samples, they were homogenised with a Precellys 24 Tissue Homogenizer (Bertin Instruments, Montigny-le-Bretonneux, France). Nucleic acid was extracted using the FastDNA Spin Kit for Soil (MP Biochemicals GmbH, Eschwege, Germany) following the manufacturer’s instructions.

### PCR amplification and illumina sequencing

PCR amplification was performed using primers targeting V3 to V4 regions of the 16S rRNA gene using the extracted DNA. Primers of 341F (5′-TCGTCGGCAGCGTC-AGATGTGTATAAGAGACAG-CCTACGGGNGGCWGCAG-3′) and 805R (5′-GTCTCGTGGGCTCGG-AGATGTGTATAAGAGACAG-GACTACHVGGGTATCTAATCC-3′) were used for amplification of the target gene. Amplification was carried out under the following conditions: initial denaturation at 95 °C for 3 min, followed by 25 cycles of denaturation at 95 °C for 30 s, primer annealing at 55 °C for 30 s, and extension at 72 °C for 30 s, with a final elongation at 72 °C for 5 min. Next, secondary amplification for attaching the Illumina NexTera barcode was performed using the i5 forward primer (5′-AATGATACGGCGACCACCGAGATCTACAC-XXXXXXXX-TCGTCGGCAGCGTC-3′; X indicates the barcode region) and i7 reverse primer (5′-CAAGCAGAAGACGGCATACGAGAT-XXXXXXXX-AGTCTCGTGGGCTCGG-3′). The conditions for secondary amplification were identical to those for the first one, except that the amplification cycle was set to 8.

The PCR product was confirmed by using 2% agarose gel electrophoresis and visualised under a Gel Doc system (BioRad, Hercules, CA, USA). The amplified products were purified with the QIAquick PCR purification kit (Qiagen, Valencia, CA, USA). Equal concentrations of purified products were pooled together and short fragments (non-target products) were removed using the Ampure beads kit (Agencourt Bioscience, Beverly, MA, USA). The quality and product size were assessed using a Bioanalyzer 2100 (Agilent, Palo Alto, CA, USA) using a DNA 7500 chip. Mixed amplicons were pooled and sequencing was carried out at ChunLab, Inc. (Seoul, Korea), using the Illumina MiSeq Sequencing system (Illumina, San Diego, CA, USA) according to the manufacturer’s instruction.

### MiSeq pipeline method

Processing of raw reads was conducted via a quality check (QC) and filtering of low quality (< Q 25) reads by Trimmomatic 0.32^51^. After the QC pass, paired-end sequence data were merged together using PandaSeq^52^. Primers were then trimmed with ChunLab’s in-house program at a similarity cut off of 0.8. Sequences were denoised using Mothur’s^53^ pre-clustering program, which merged sequences and extracted unique sequences allowing up to 2 differences between sequences. The EzTaxon database was used for taxonomic assignment using BLAST 2.2.22 and pairwise alignment was used to calculate similarity^54,55^. Uchime and the non-chimeric 16S rRNA database from EzTaxon were used to detect chimeras on reads that had a best hit similarity rate of less than 97%^56^. Sequence data were then clustered using CD-Hit and UCLUST, and alpha diversity analysis was carried out^57^. It is to be noted that in this database, the uncultured phylotype is tentatively given the hierarchal name assigned to the DDBJ/ENA/GenBank accession number with the following suffixes: “_s” (for species), “_g” (genus), “_f” (family), “_p” (phylum)^58^.

### Diversity analysis

Alpha diversity with Simpson index was used to compare gut microbial diversities between group of collected honey bee, and the *p* value was calculated with Wilcoxon t-test. The difference of gut bacterial community between SBV-resistant and -susceptible honey bee strain was also determined by NMDS distancing by Bray–Curtis index. The analysis was done by using Vegan community ecology package version 2.5-7, and visualized by using ggplot2 package version 3.3.5.

### Statistical analysis

Comparison of microbiota in larvae with SD to larvae without SD, in adult with SD to adult without SD, in SBV-resistant larvae to SBV-susceptible larvae, in resistant adult to susceptible adult, and the abundance of different microbiotic strains in different seasons and different regions was done using Mann–Whitney U tests (non-parametric) from program PAST version 4.03. The differences between samples were considered to be significant when *p* < 0.05.

## Supplementary Information

Supplementary Figures.

Supplementary Tables.

## Data Availability

All data generated or analysed during this study are included in this published article and its Supplementary Information files.
