# Supplementary material for: Comparison of the gut microbiome of sacbrood virus-resistant and -susceptible Apis cerana from South Korea
Source: Sci Rep. 2022 Jun 15;12:10010. doi: 10.1038/s41598-022-13535-0 (PMC9200864; doi:10.1038/s41598-022-13535-0)
Supplement: Supplementary file 1 — Supplementary Figures. [file 41598_2022_13535_MOESM1_ESM.docx]

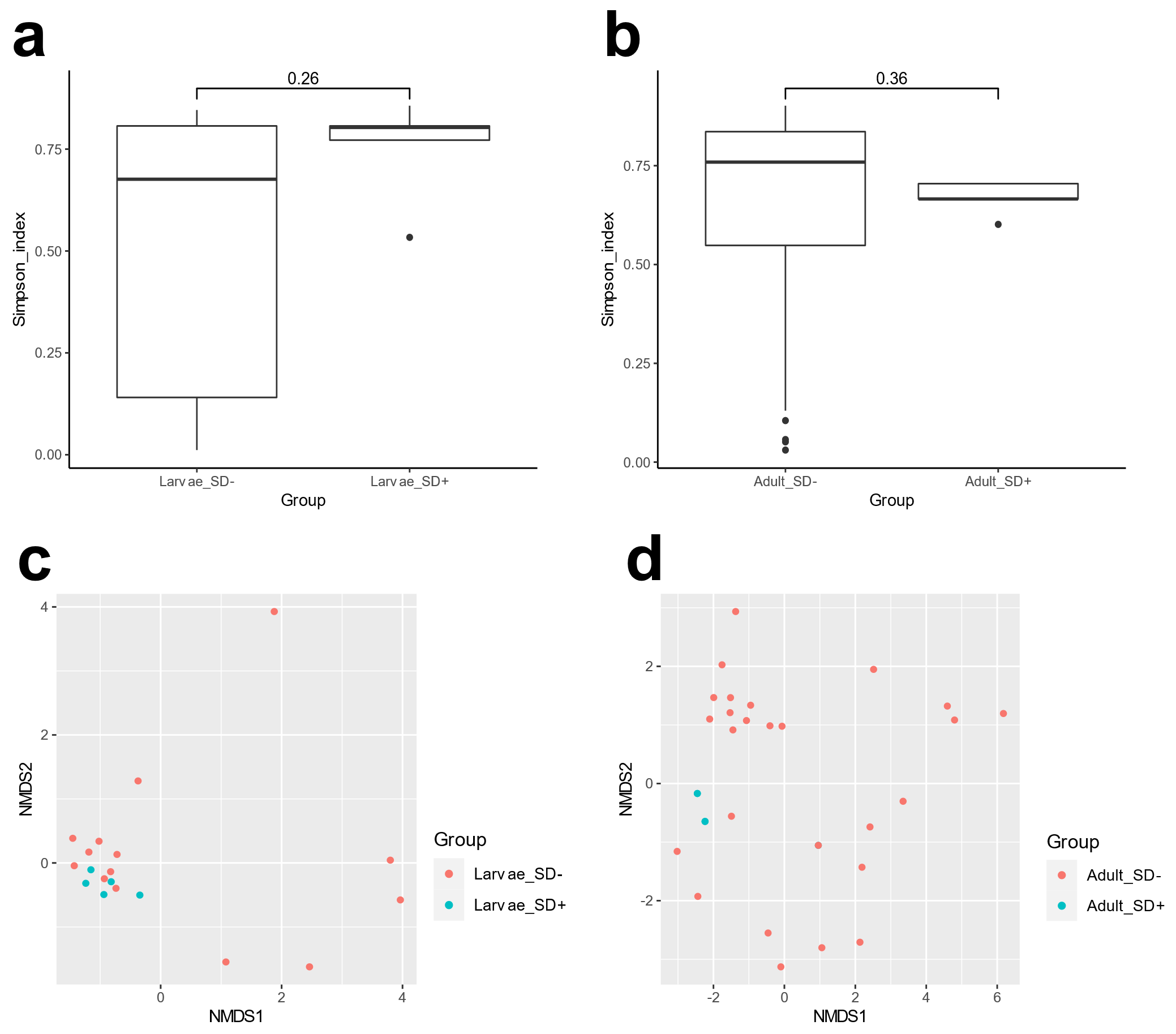


**Figure S1. Comparison of gut microbiome between SBV free (SD-) and SBV disease (SD+) samples of susceptible *A. cerana*.** Alpha diversity with Simpson index was performed to compare gut microbiome of larval samples (a) and adult samples (b), the *p* values calculated with Wilcoxon t-test are shown. The non-metric multidimensional scaling (NMDS) distancing by Bray-Curtis index was also done for comparison of microbiome of larval samples (c) and adult samples (d), the stress values were 0.0889 and 0.1478, respectively.


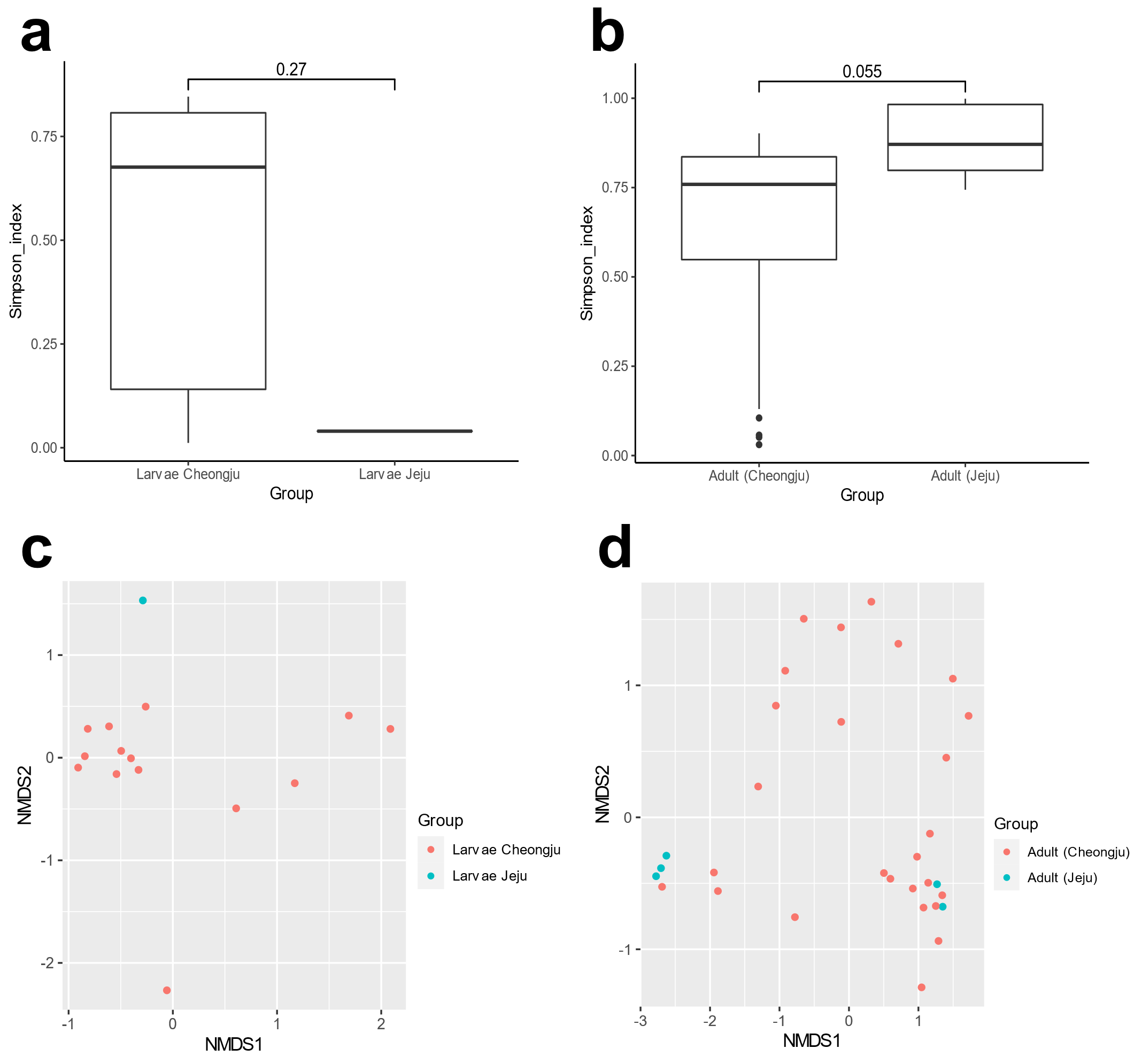


**Figure S2. Comparison of gut microbiome of SBV susceptible *A. cerana* collected from Cheongju and Jeju province.** Alpha diversity with Simpson index was performed to compare gut microbiome of larval samples (a) and adult samples (b), the *p* values calculated with Wilcoxon t-test are shown. The non-metric multidimensional scaling (NMDS) distancing by Bray-Curtis index was also done for comparison of microbiome of larval samples (c) and adult samples (d), the stress values were 0.0990 and 0.1290, respectively.


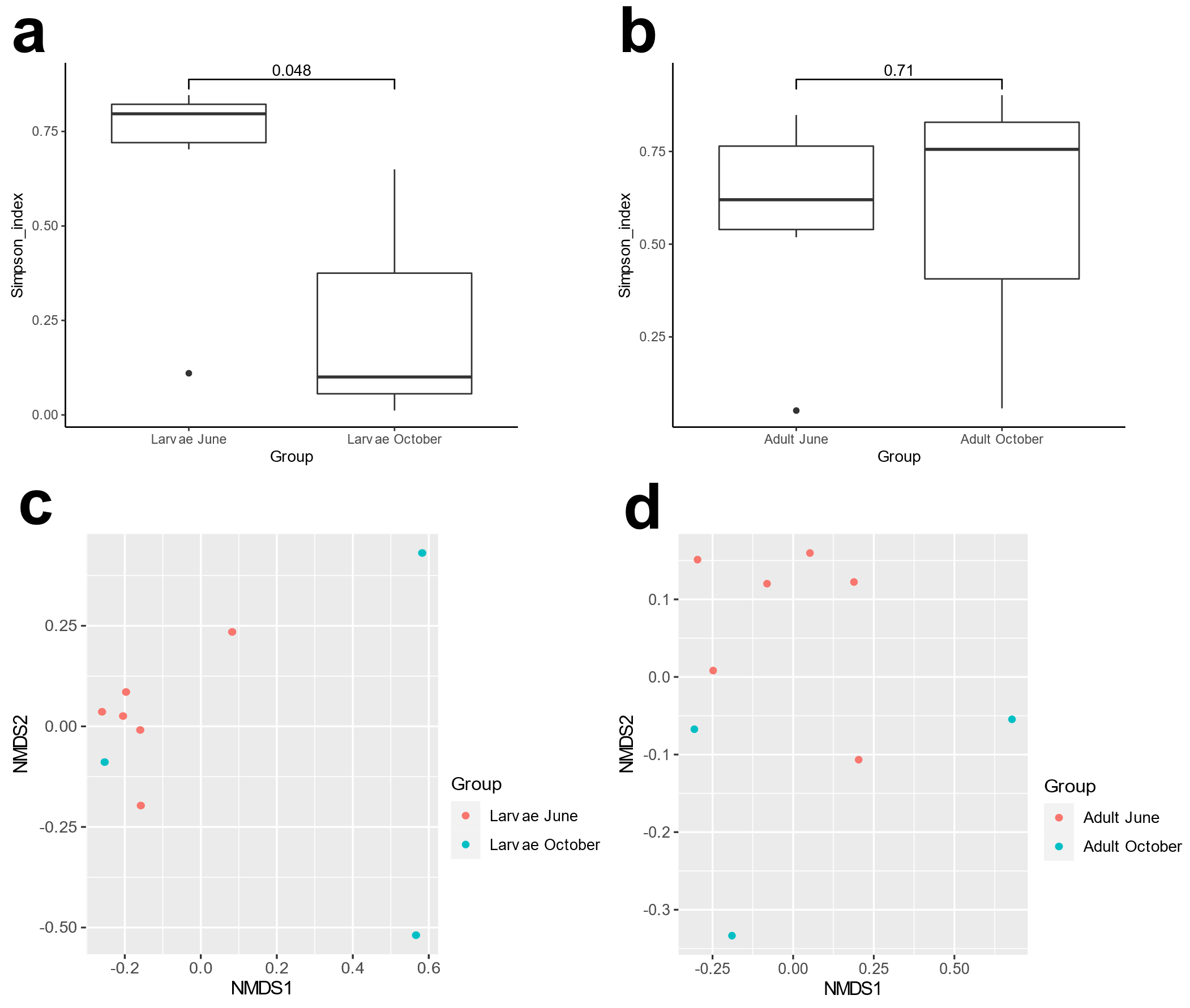


**Figure S3. Comparison of gut microbiome of SBV susceptible *A. cerana* collected in June and October from Cheongju province.** Alpha diversity with Simpson index was performed to compare gut microbiome of larval samples (a) and adult samples (b), the *p* values calculated with Wilcoxon t-test are shown. The non-metric multidimensional scaling (NMDS) distancing by Bray-Curtis index was also done for comparison of microbiome of larval samples (c) and adult samples (d), the stress values were 0.0524 and 0.0638, respectively.


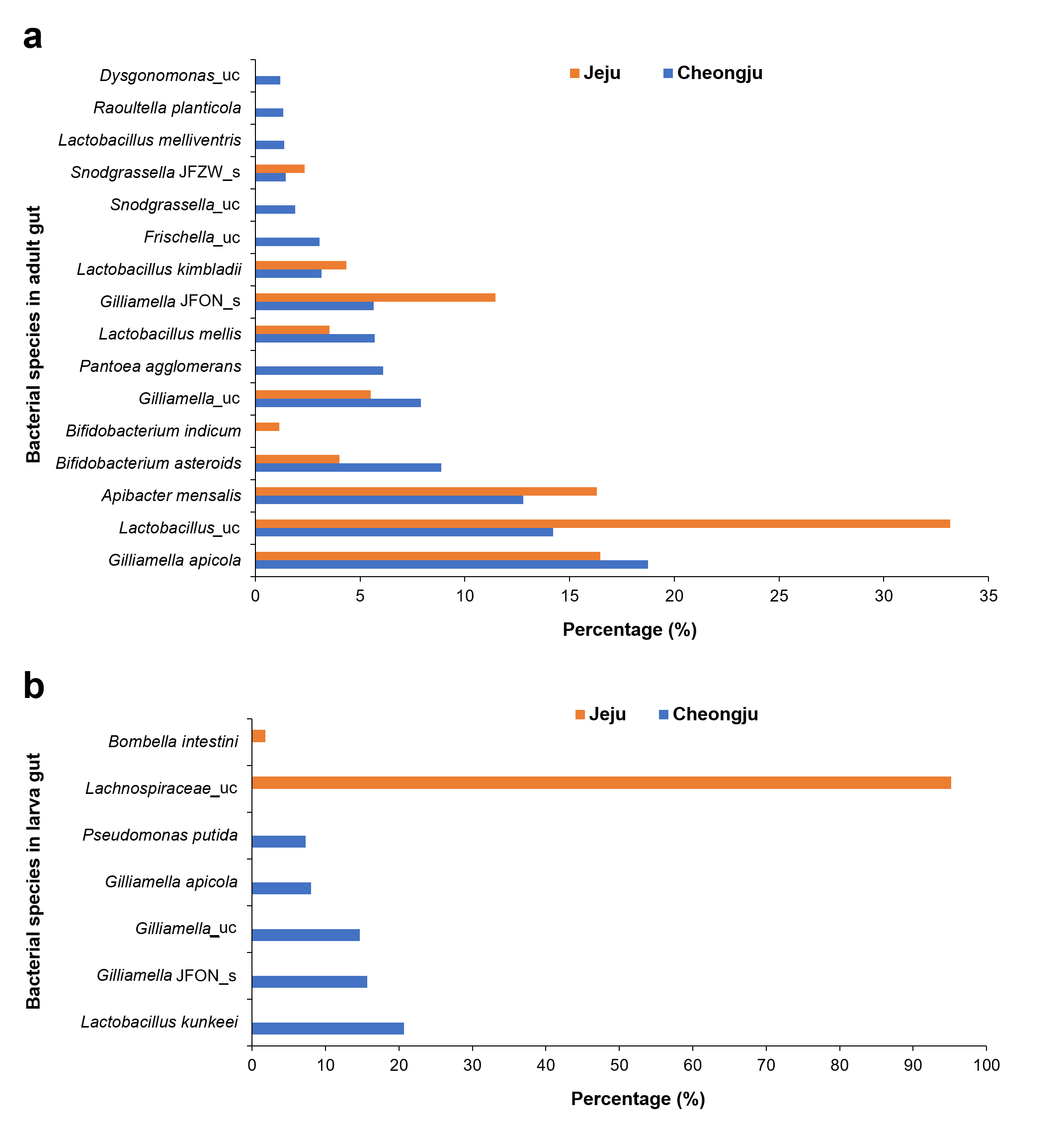


**Figure S4. Gut microbiome of *A. cerana* collected from different regions.** The gut microbiota of healthy individuals of SBV-susceptible **(a)** adults and **(b)** larvae, collected from Jeju and Cheongju province in June, was identified. (“uc” indicates unclassified species; “JFON_s” and “JFZW_s” indicate uncultured species of genera *Gilliamella* and *Snodgrassella*, respectively.)


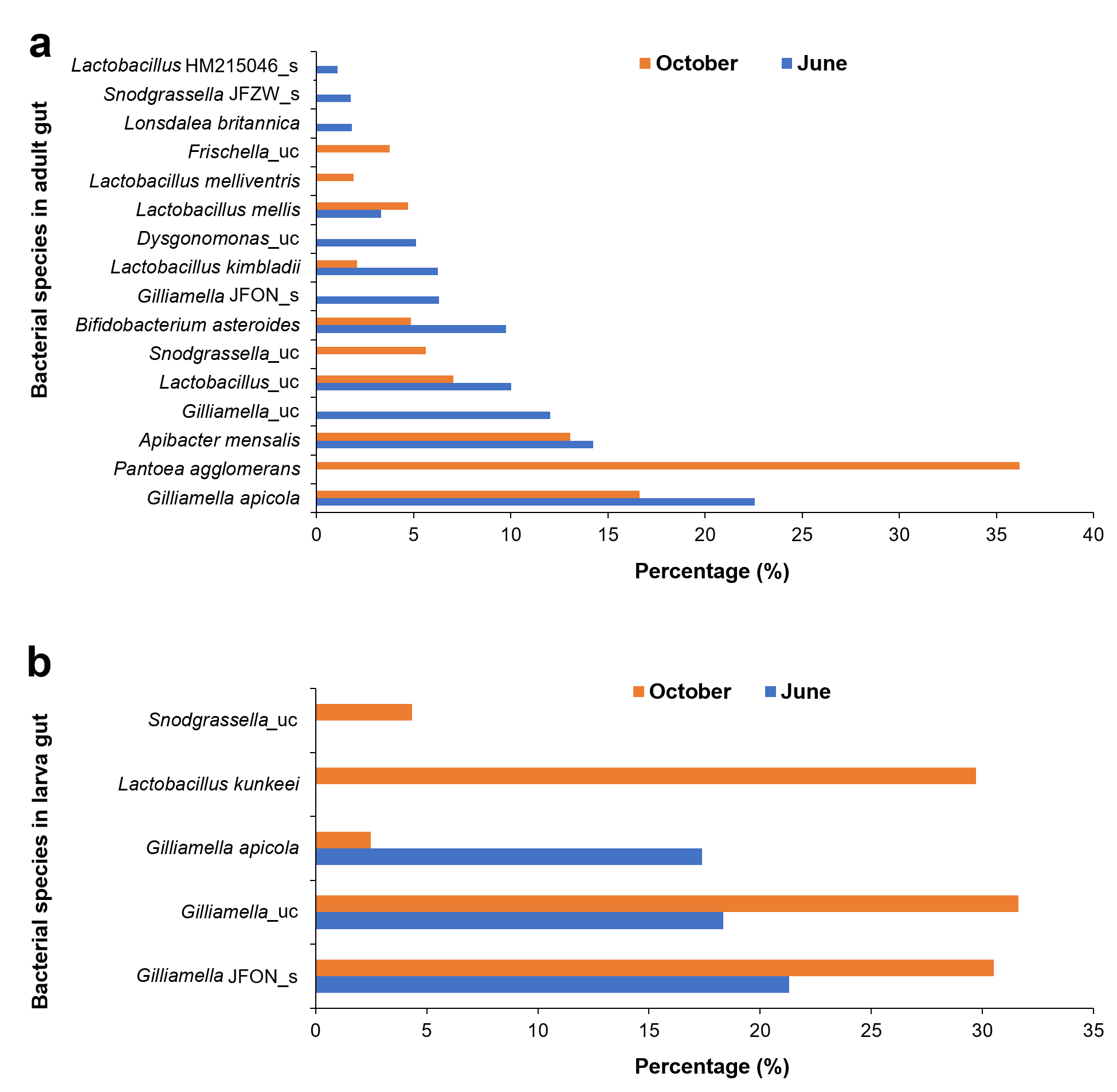


**Figure S5. Gut microbiome of *A. cerana* collected in different seasons.** The gut microbiota of healthy individuals of SBV-susceptible **(a)** adults and **(b)** larvae, collected from Cheongju province in June and October, was identified. (“uc” indicates unclassified species; “JFON_s” and “JFZW_s” indicate uncultured species of genera *Gilliamella* and *Snodgrassella*, respectively.)
