# Supplementary material for: Comparison of the gut microbiome of sacbrood virus-resistant and -susceptible Apis cerana from South Korea
Source: Sci Rep. 2022 Jun 15;12:10010. doi: 10.1038/s41598-022-13535-0 (PMC9200864; doi:10.1038/s41598-022-13535-0)
Supplement: Supplementary file 2 — Supplementary Tables. [file 41598_2022_13535_MOESM2_ESM.docx]

**Table S1. Gut microbiota identified from *Apis cerana***

| Phylum | Species | SBV-susceptible larvae | | SBV-resistant larvae | | SBV-susceptible adult | | SBV-resistant adult | |
| --- | --- | --- | --- | --- | --- | --- | --- | --- | --- |
|  |  | **(-)** | **(+)** | **R** | **H** | **(-)** | **(+)** | **R** | **H** |
| Proteobacteria | *Bombella intestini* group | - | - | - | 3.11% | - | - | - | - |
|  | *Frischella_*uc | - | - | - | - | 1.97% | - | - | - |
|  | *Gilliamella apicola* group | 7.51% | 98.20% | 7.38% | - | 18.35% | 20.55% | 9.98% | 8.71% |
|  | *Gilliamella* JFON_s | 14.81% | - | - | - | 6.37% | 23.99% | 6.45% | 17.01% |
|  | *Gilliamella_*uc | 13.71% | - | - | - | 7.53% | 8.20% | 8.48% | 17.66% |
|  | *Hafnia alvei* group | - | - | - | - | - | - | 15.81% | 5.26% |
|  | *Pantoea agglomerans* group | - | - | - | - | 5.25% | - | - | - |
|  | *Pseudomonas putida* group | 6.81% | - | - | - | - | - | - | - |
|  | *Snodgrassella* JFZW_s | - | - | - | - | 1.84% | 1.29% | 1.07% | 2.88% |
|  | *Snodgrassella_*uc | - | - | - | - | 1.73% | - | - | - |
| Firmicutes | *Lactobacillus kimbladii* | - | - | - | - | 3.27% | 6.03% | 3.32% | 2.08% |
|  | *Lactobacillus kunkeei* group | 19.29% | - | - | - | - | - | - | - |
|  | *Lactobacillus mellifer* | - | - | - | - | - | - | 1.48% | 1.30% |
|  | *Lactobacillus mellis* | - | - | - | - | 5.35% | 4.44% | 3.09% | 3.95% |
|  | *Lactobacillus melliventris* | - | - | - | - | - | 1.06% | - | - |
|  | *Lactobacillus* HM215046_s | - | - | - | - | - | - | 1.22% | 1.59% |
|  | *Lactobacillus_*uc | - | - | 1.50% | - | 17.14% | 13.52% | 23.46% | 14.13% |
|  | *Melissococcus plutonius* | - | - | - | - | - | - | - | - |
| Bacteroidetes | *Apibacter mensalis* | - | - | - | - | 13.24% | 16.47% | 17.13% | 15.19% |
| Actinobacteria | *Bifidobacterium asteroides* group | - | - | - | - | 8.08% | 3.02% | 5.04% | 6.60% |
| Unclassified | Unclassified | 30.49% | - | 76.13% | 22.61 | - | - | - | - |

Note: cut-off value was 1%; (-) and (+) denote for healthy and SD honeybees, respectively. “-” was no information.

**Table S2. Lactic acid bacteria identified from gut of *Apis cerana***

| Phylum | Species | SBV-susceptible larvae |  | SBV-resistant larvae |  | SBV-susceptible adult |  | SBV-resistant adult |  |
| --- | --- | --- | --- | --- | --- | --- | --- | --- | --- |
|  |  | (-) | (+) | R | H | (-) | (+) | R | H |
| *Actinobacteria* | *Bifidobacterium asteroides* group | 0.108% | - | 0.323% | 0.022% | 8.077% | 3.023% | 5.040% | 6.598% |
|  | *Bifidobacterium bifidum* | 0.048% | - | - | 0.016% | - | - | - | - |
|  | *Bifidobacterium catenulatum* group | - | - | - | 0.021% | - | - | - | - |
|  | *Bifidobacterium indicum* group | - | - | - | - | - | - | 0.013% | 0.084% |
|  | *Bifidobacterium longum* group | - | - | 0.013% | 0.060% | 0.367% |  |  |  |
| *Firmicutes* | *Lactobacillus algidus* | 0.051% | - | 0.083% | - | - | - | - | - |
|  | *Lactobacillus apis* | - | - | 0.035% |  | 0.052% | 0.077% | 0.073% | 0.019% |
|  | *Lactobacillus gasseri* group | 0.074% | - | - | - | - | - | - | - |
|  | *Lactobacillus helsingborgensis* | 0.036% | - | 0.057% |  | 0.498% | 0.175% | 0.586% | 0.937% |
|  | *Lactobacillus helveticus* group | 0.010% | - | - | - | - | - | - | - |
|  | *Lactobacillus kimbladii* | 0.122% | 0.022% | 0.458% | 0.012% | 3.274% | 6.025% | 3.317% | 2.076% |
|  | *Lactobacillus kullabergensis* | - | - | - | - | 0.026% | - | 0.011% | - |
|  | *Lactobacillus kunkeei* group | 19.293% | - | - | - | 0.077% | - | - | - |
|  | *Lactobacillus mellifer* | - | - | 0.042% | - | 0.395% | 0.011% | 1.483% | 1.303% |
|  | *Lactobacillus mellis* | 0.063% | - | 0.211% | 0.014% | 5.348% | 4.440% | 3.087% | 3.954% |
|  | *Lactobacillus melliventris* | - | - | 0.033% | - | 0.982% | 1.590% | 0.028% | 0.084% |
|  | *Lactobacillus murinus* group | 0.125% | - | - | - | - | - | - | - |
|  | *Lactobacillus plantarum* group | 0.032% | - | - | 0.025% |  |  |  |  |
|  | *Lactobacillus reuteri* group | 0.050% | - | - | - | - | - | - | - |
|  | *Lactobacillus sakei* group | 0.097% | - | 0.054% |  |  |  |  |  |
|  | *Lactobacillus salivarius* | - | - | - | - | - | - | - | - |
|  | *Lactococcus lactis* group | - | - | 0.011% | - | - | - | - | - |
|  | *Leuconostoc mesenteroides* group | - | - | 0.038% | - | 0.025% |  |  |  |
|  | *Weissella kandleri* group | 0.048% | - | 0.139% | - | - | - | - | - |

Cut-off value: 0.01%; (-) and (+) denote for the negative and positive infection of SBV, respectively. “-” was no information.
